# Editorial: Machine Learning Techniques for Soft Robots

**DOI:** 10.3389/frobt.2021.726774

**Published:** 2021-07-01

**Authors:** Thomas George Thuruthel, Egidio Falotico, Lucia Beccai, Fumiya Iida

**Affiliations:** ^1^ The Bio-Inspired Robotics Lab, Department of Engineering, University of Cambridge, Cambridge, United Kingdom; ^2^ The BioRobotics Institute, Scuola Superiore Sant’Anna, Pontedera, Italy; ^3^ Department of Excellence in Robotics and AI, Scuola Superiore Sant’Anna, Pisa, Italy; ^4^ Soft BioRobotics Perception Lab, Istituto Italiano di Tecnologia, Genova, Italy

**Keywords:** soft robotics, machine learning, editorial, soft sensing, modeling and control

Soft robotic technologies have introduced new paradigms in the design and development of robots. This shift in outlook presents new challenges and opportunities for modeling, control, and design of these robots. Traditional techniques based on analytical models have proven to be insufficient to tackle these new challenges. This is because of their highly nonlinear, time-varying and high-dimensional characteristics coupled with an immense diversity in their design. Machine learning-based approaches provide a promising alternative to traditional analytical approaches. Learning-based approaches have proven to be a valuable tool for control, data-processing, and design optimization of nonlinear systems in traditional robotics and other scientific disciplines. However, their usage has been largely limited and unexplored in soft robotics, in spite of their potential value.

This research topic was initiated to investigate and advance new learning-based approaches for modeling, control, and design of soft robots. The eight articles present novel approaches for modeling, sensing and design optimization. When it comes to modeling soft robotic systems it can be clearly seen that the field is turning toward recurrent neural networks (RNNs) (Tariverdi et al., 2021; Tsompanas et al., 2021) or hybrid approaches (Johnson et al., 2021). In order to capture the temporal nonlinearities in a soft robotic system, it is vital to use learning architectures that have dynamic properties. This was demonstrated for real-time dynamic modeling of a soft continuum manipulator in Tariverdi et al. (2021) and for characterization of a Microbial Fuel Cell in Tsompanas et al. (2021). Hybrid models combining physics-based analytical models and deep learning also promises to be an alternate approach, especially when data is scarce as shown in Johnson et al. (2021).

A significant portion of the submitted works focus on sensing and state estimation for soft robots, a topic which has wider applications in wearables and biomedical fields. De Barrie et al. (2021) presented a learning based framework for contact force prediction and stress distribution in real-time using deep learning and FEA models. Such techniques are powerful tools to reduce computational complexity without compromising on accuracy. Hofer et al. (2021) presented a vision-based sensing approach for state estimation in soft robots using convolutional neural networks. Khin et al. (2021) developed grip-state estimation networks for feedback control of sensorized soft robotic hands. Finally, Raffin et al. (2021) presented ensemble networks to detect and handle sensor failures.

Design optimization is one of the biggest challenge in soft robotics due to the computational complexity of parametrized analytical models. Raeisinezhad et al. (2021) presented a deep reinforcement learning (DRL) algorithm for design optimization of a pneumatic soft robotic actuator on a simulated model. The key idea here being that DRL methods would be more sample efficient than traditional optimization methods with additional capabilities. In conclusion, learning-based techniques hold a strong potential for addressing the major challenges in soft robotics, be it design, modeling, sensing or control. Different learning architectures (recurrent neural networks, deep neural networks, hybrid networks) and approaches (supervised/unsupervized learning, reinforcement learning) have to be adopted based on the problem and application. Finally, machine learning can become a vital tool when used in soft sensing. In particular to obtain repeatable and reliable data in spite of non-linearities of constituting materials and/or sensor failures.
